# Perceived social support and self-efficacy beliefs for healthy eating and physical activity among Arabic-speaking university students: adaptation and implementation of health beliefs survey questionnaire

**DOI:** 10.1186/s12889-021-12138-0

**Published:** 2021-11-09

**Authors:** Habiba I. Ali, Salma Alhebshi, Fadima Elmi, Mo’ath F. Bataineh

**Affiliations:** 1 Department of Nutrition and Health, College of Medicine and Health Sciences, United Arab Emirates University, P.O. Box 15551, Al Ain, United Arab Emirates; 2 Zayed Center for Health Sciences, United Arab Emirates University, Al Ain, United Arab Emirates; 3 Independent Researcher, P.O. Box 67258, Al Ain, United Arab Emirates; 4 Departement of Sport Rehabilitation, The Hashemite University, Zarqa, Jordan

**Keywords:** Arabic-speaking, Cultural adaptation, Social support, Self-efficacy university students

## Abstract

**Background:**

Few assessment tools exist for investigating perceived social support and self-efficacy behaviors in Arabic-speaking populations. Moreover, literature on the levels of social support and self-efficacy for adopting healthy eating and engaging in regular physical activity among Arabic-speaking young adults is currently lacking. This study aimed to adapt the Health Beliefs Survey Questionnaire (HBSQ) for Arabic-speaking populations and assess perceived social support and self-efficacy for adopting healthy eating and increased physical activity among university students.

**Methods:**

In the first stage of the study, forward and backward translation and pretesting of the social support and self-efficacy scales of the HBSQ were conducted. The adapted questionnaire was administered to female university students (*n* = 258), and a subsample of 195 participants retook the questionnaire after 1 month. Construct validity was assessed with confirmatory factor analysis. The internal consistency of each subscale item was assessed by Cronbach’s alpha coefficient, and reproducibility was tested with intraclass correlation coefficients (ICCs) and Bland-Altman plots. In the second stage, the adapted questionnaire was used to assess the perceived social support and self-efficacy levels in a different sample of Arabic-speaking female university students (*n* = 283).

**Results:**

Based on the results from confirmatory factor analysis, 6 items were selected for the social support scale and 19 items for the self-efficacy scale. The adapted questionnaire showed moderate to high internal reliability (Cronbach alpha coefficient = 0.681 to 0.900). The ICCs of the various subscales ranged from 0.666 to 0.997, indicating moderate to excellent reproducibility of the culturally adapted questionnaire. This was confirmed by Bland-Altman analysis. Participants in the second stage of the study reported significantly higher (*P* < 0.001) perceived support from family compared to friends in reducing sugar intake and increasing fiber consumption.

**Conclusions:**

The results of the psychometric testing indicate the shortened Arabic HBSQ is a reliable tool for assessing perceived social support from family and close friends as well as for evaluating self-efficacy for choosing healthy foods and increasing physical activity among female university students.

## Background

In the United Arab Emirates (UAE), overweight, obesity, and associated chronic diseases have emerged as major public health concerns among all age groups [1–3]. A nationally representative survey of 628 randomly selected Emirati households in all seven emirates found that 65% of adult women (19–50 yrs), 28% of male adolescents and 40% of female adolescents were either overweight or obese [4]. A recent shift has occurred in the country toward habits such as the consumption of fast foods and high-energy foods that are easy to obtain [4].

Healthy dietary patterns are crucial for reducing the risk of noncommunicable chronic diseases. An assessment of diet quality among adults in 187 countries found the lowest diet quality score among young adults aged 20–29 years compared to any other age group [5]. The food choices of young adults, such as university students, are most likely not to meet current recommendations due to changes associated with moving away from home, busy schedules, and unhealthy eating patterns [6–8]. A previous study involving 400 female university students in the UAE reported that 19.4% were overweight, 6.7% were obese, 45% skipped breakfast, and 35% ate fast food at least once daily [9]. The results from a recent study involving 251 female university students in the UAE [10] showed a high prevalence of overweight, obesity and anemia (17.4, 11.9, and 18.1%, respectively). In 2014, 42.2% of male and 21.3% of female university students were either overweight or obese [11]. A cross-sectional study involving 308 university students found that the majority of the participants had poor body image perception [12]. Moreover, a heavy consumption of added sugars and low nutrition knowledge was reported among college students in the UAE [13]. Understanding the factors influencing food choice is critical in developing effective health promotion strategies. The role of social support from family and friends is known to be particularly important in helping people adopt healthier lifestyles [14]. A review of 34 articles that assessed barriers and enablers of healthy eating among young adults found that one of the main enablers was having friends and family members with healthy eating behaviors [15]. This may have specific relevance to the UAE community due to strong cultural traditions such as eating together with family and friends. On the other hand, a barrier to adopting healthy food choices was having friends and family members with unhealthy diets. In a previous study, university students in the UAE identified friends as one of the main influencing factors in choosing unhealthy foods from campus-based vending machines [16].

Social cognitive theory (SCT) explains the relationship between personal factors (e.g., self-efficacy), environmental factors,and behavior (e.g., choosing healthy foods) [17]. It is a well-recognized tool for identifying methods in which behaviors can be modified or changed [18–21]. Two major SCT constructs that can be applied to adopting healthy eating and regular physical activity are self-efficacy and social support. **Self-efficacy** refers to an individual’s belief in his or her capacity to execute behaviors necessary to produce specific performance attainments [17, 22, 23]. **Social support** is support from close relations, such as family and friends, and it can enhance self-efficacy in SCT. In social support, peers, friends, neighbors and colleagues can all play a role in engaging in healthy behaviors [24, 25]. Perceived support from family and friends has been shown to be an important predictor of nutrition and health-related behaviors [26, 27].

The Health Beliefs Survey Questionnaire (HBSQ) is a multidimensional psychosocial questionnaire based on SCT developed by Anderson et al. [18, 28]. It is used to assess important constructs that influence behavior, including self-efficacy and social support beliefs related to choosing healthy foods and engaging in physical activity [18, 28]. The validity and reliability of the HBSQ were tested among a sample of adults in the United States [18, 29]. The questionnaire was found to be an important predictor of nutrition and physical activity [30].

Although SCT constructs such as self-efficacy and social support play an important role in modifying health behaviors, to our knowledge, culturally appropriate assessment tools for social support and self-efficacy related to eating and physical activity behaviors among Arabic-speaking populations are currently lacking. Such assessment tools are critical in understanding the psychosocial determinants of food and physical activity behaviors among Arabic-speaking young adults. The objectives of the current study were (1) to adapt the social support and self-efficacy scales of an SCT-based questionnaire, the HBSQ, for Arabic-speaking university students and (2) to assess the perceived social support and self-efficacy for adopting healthy eating and increased physical activity among university students using the adapted HBSQ.

## Methods

### Study design and participants

This study was designed to adapt the HBSQ for Arabic-speaking university students and assess their perceived social support and self-efficacy for eating healthy foods and regular physical activity. In the first stage of the study, we adapted the social support and self-efficacy scales of the HBSQ to the Arabic language using the forward/backward translation method and conducted psychometric testing of the translated questionnaire.

After forward/backward translation and pretesting, the questionnaire was reproduced in Google forms and was completed by 258 female university students. Participants were recruited from a major university in the UAE. Eligible participants were required to be female and between the ages of 18 and 35 years old. These eligibility criteria were chosen since the questionnaire, once adapted, was intended for use in a study involving lifestyle intervention program for university students. A subsample of 195 participants completed the questionnaire after 1 month. The adapted questionnaire was then used to assess the perceived social support and self-efficacy levels in another sample of 283 Arabic-speaking female university students.

To recruit participants for both stages of the study and to maximize the exposure of the program, a poster advertising the study was displayed in a number of areas of the campus frequented by students, including the food court, residential halls, and the health club. The poster was also distributed via social media platforms, including WhatsApp and the university students’ Facebook and Instagram accounts.

### The instrument: the HBSQ

The validity and reliability of the original English version of the HBSQ were established by Anderson and colleagues [18, 28, 31]. The questionnaire consists of 4 scales (social support, self-efficacy, self-regulation strategies, and outcome expectations). Each scale is divided into two main measures: healthy eating and physical activity. In this study, we used the social support and self-efficacy scales. The social support subscale is a measure of social influences on participants by their family members and closest friends, Examples of the questions in this subscale are as follows: to assess social support to reduce fat consumption participants were asked, using a scale from 1 (strongly disagree) to 5 (strongly agree), tell us if you agree with the following statement: *“My Family/My closest Friends have told me they want to cut down on sweets”* and to assess social support for increasing fiber, fruit and vegetable intake participants were asked: using a scale from 1 (strongly disagree) to 5 (strongly agree), tell us if you agree with the following statement: *“My Family/My closest Friends have told me they want to eat more fruits and vegetables”.*

The healthy food efficacy measure assesses the respondent’s confidence in reducing fat, reducing sugar consumption and increasing fruit and vegetable intake. An example of a question in this measure is as follows: Using a scale of 0–100, where 0 is certainly I cannot, 50 is somewhat I can and 100 is certainly I can, *“How certain are you that you can bring a fruit to work or school for a snack every day?”*

The physical activity measure consists of two subscales: overcoming barriers to engaging in physical activity and integrating physical activity into the daily routine. The first subscale assesses the respondent’s confidence in overcoming specific barriers all or most of the time. For example, it includes items that ask the following: Use any number from 0 to 100 to tell how certain you are that you can – all or most of the time; *“How certain are you that you can begin increasing your step count again if you miss a day or two?”* An example of the integrating physical activity is as follows: Use any number from 0 to 100 to tell how certain you are that you can – all or most of the time; *“How certain are you that you can use the stairs at work or school instead of the elevator?”* If participants were more certain regarding integrating physical activity into their daily routine than overcoming barriers, it would indicate that interventions should target strategies that assist the target population in overcoming barriers to regular physical activity.

### Stage 1: adaptation of the HBSQ for Arabic-speaking populations

To adopt the previously validated HBSQ concerning social support and self-efficacy beliefs related to healthy food consumption and physical activity [18, 28], we conducted a linguistic adaptation of it for Arabic-speaking populations and evaluated our adapted questionnaire’s reliability and reproducibility. We followed a series of steps according to the translation and adaptation of instruments developed by the World Health Organization (WHO) [32] to adapt the perceived social support and self-efficacy scales for healthy eating and physical activity of the original questionnaire for Arabic-speaking university students.

- Step 1: Three native Arabic language speakers translated the English version into Arabic (forward translation).
- Step 2: Two nutritionists working in an academic setting reviewed the draft translation.
- Step 3: Two bilingual academic instructors and two nutritionists independently reviewed the Arabic translations against the original questionnaire to ensure that the meaning of the items in the original questionnaire were retained.
- Step 4: Back translation of the Arabic version to English was performed by two translation specialists.
- Step 5: Cognitive debriefing interviews with 15 university students from different Arabic-speaking countries were performed. This step was conducted to test the comprehension and clarity of the questionnaire by native Arabic speakers from diverse countries. The students confirmed that both the items and the instructions were clear and that the language was simple. Minor changes were made to the questionnaire based on feedback from the cognitive debriefing. For example, the food item “Melba toast” in the efficacy scale was changed to “Shabora bread”, which is a more common food item in Arabic communities and has similar nutritional value.
- Step 6: The questionnaire was pilot tested with 30 university students. The participants were asked to comment on the clarity and comprehension of the questionnaire and whether any additional explanations were needed for the questions in the scales and the instructions. There were no additional modifications suggested by the pilot participants.
- Step 7: The adapted questionnaire was administered to 258 female university students.

Construct validity of the Arabic version of the questionnaire was tested using confirmatory factor analysis and reliability using Cronbach’s alpha. Reproducibility after 1-month was tested using intraclass correlations.

In the second stage of the study, the adapted Arabic social support and self-efficacy scales were administered to 283 female university students to assess their perceived levels of social support and self-efficacy for adopting healthy eating and regular physical activity.

### Anthropometric measurements

An appointment was made for participants who were interested in participating in the second stage of the study to attend the university nutrition clinic for anthropometric measurements. Height, weight and body composition (percent body fat and lean mass) were measured using a composition analyzer (TANITA®-BC420MA) following the manufacturer’s instructions. Before completing the questionnaires, body mass index was calculated to exclude students who were underweight. We included only participants who were overweight, obese or within healthy weight to determine if differences in the perceived social support and self-efficacy exist between students who are within healthy weight and those who are either overweight or obese.

### Statistical analysis

During the first stage of the study, descriptive statistics were conducted for each item and skewness and kurtosis were calculated. The fit of the model to the data was assessed using Confirmatory Factor Analysis (CFA). The CFA was conducted using maximum likelihood (ML) estimation and bootstrapping (*n* = 1000 simulated samples) to achieve full use of available data and to manage normality issues. Initially, CFA was used to obtain standardized factor loading values and items with standardized loading values of less than 0.6 were deleted to improve model fit. Various types of model fit indices were used including the Chi-square (good fit: *p*-value > 0.05), Chi-square/degrees of freedom (*χ*^2^/df: good fit ≤3), Root Mean Square Error of Approximation (RMSEA: good fit < 0.060), Comparative Fit Index (CFI: good fit > 0.950), and Tucker-Lewis Index (TLI: good fit > 0.900) [33]. The CFA was conducted using JASP version 0.14.1 (University of Amsterdam, Amsterdam, The Netherlands).

The internal consistency of the items in each subscale was assessed using Cronbach’s alpha. Reproducibility was assessed using intraclass coefficient correlations (ICC) to compare the scores of the responses obtained after administration of the questionnaire twice. Bland-Altman plots were performed to confirm and visually examine the agreement of the scores of each subscale between the first and second administration of the questionnaire. The 95% limits of agreement (LOA) and 95% confidence interval (CI) were calculated. Minitab 18 was used to create the Bland-Altman plots. Data obtained from Stage 2 was analyzed using paired and independent sample t-tests to assess the levels of social support and self-efficacy of the participants. Cronbach’s alpha, ICC and t-tests were performed using Statistical Package for Social Science, SPSS (version 25). *p*-values < 0.05 were considered statistically significant.

### Ethical considerations

This study was approved by the United Arab Emirates University Human Research Ethics Committee (protocol # 33; 2014/2015). Participants were informed that their participation in the project was voluntary and that all information collected would remain anonymous and confidential. Verbal and written informed consent were obtained from all participants prior to data collection. All methods of the study were carried out in accordance with the guidelines and regulations of the United Arab Emirates University Human Research Ethics Committee.

## Results

### Stage 1. Adaptation of HBSQ

#### Construct validity and reliability of the Arabic version of HBSQ

In the first stage of the study, we recruited 258 female university students to test the construct validity and reliability of the of the social support and self-efficacy scales of the HBSQ for Arabic-speaking university students. The mean age (SD) of the participants was 21.4 (1.85) years. The majority (98.8%) were undergraduate students, and the remaining were graduate students. Regarding the nationality of the participants, 79.5% were UAE nationals (Emirati), and the remaining 20.5% were other Arabs.

The standardized item loading values generated from Confirmatory Factor Analysis (CFA) with the use of maximum likelihood (ML) estimations for the model fit of the revised social support scale is shown in Table 1. The resulting model consisted of 3 factors and 6 items. The current model showed a good fit across all indices (*χ*^2^/df = 2.2; RMSEA = 0.052 (90% CI 0.031–0.068); CFI = 0.976; and TLI = 0.954) except a significant Chi-square (*χ*^2^ (11) = 24.185, *p*-value = 0.012). The reliability analysis results of the social support scale and subscales of the HBSQ adapted for Arabic-speaking young adults are shown in Table 1, the Cronbach alpha values showed adequate (moderate to high) internal consistency overall and across all the subscales.

**Table 1:** Perceived social support subscales in the Arabic adaptation of the health beliefs survey Questionnaire: Construct validity and internal consistency estimates (*n* = 285)^**a**^

Item	Mean	SD	α	Skewness	Kurtosis	Item Loading
**Overall**			0.737			
**Increase fiber intake**			0.771			
Family/friends eat higher-fiber bread every day	3.03	1.430		−0.271	−1.344	0.753
Family/friends have told me they want to eat higher-fiber bread.	3.42	1.359		−0.444	−1.015	0.833
**Decrease fat intake**			0.758			
Family/friends have told me they want to cut down on high-fat dairy foods	3.21	1.313		−0.192	−1.048	0.676
Family/friends try to eat low-fat dairy foods	3.24	1.262		− 0.286	−0.919	0.852
**Increase physical activity**			0.885			
Family/friends find or hire a babysitter so they can increase their physical activity	2.10	1.216		0.831	−0.337	0.842
Family/friends are not more physically active because they get too hot	2.15	1.240		0.808	−0.364	0.944

^**a**^Perceived social Support (Family and Friends) for healthier foods and physical activity subscales (1 - Strongly disagree, 5 - Strongly agree)

α - Cronbach’s alpha coefficient; item loading – factor loading from Confirmatory Factor Analysis.

The standardized item loading values generated by conducting Confirmatory Factor Analysis (CFA) with the use of maximum likelihood (ML) estimations for the model fit of the revised self-efficacy scale are shown in Table 2. The resulting model contained 6 factors and 19 items. The model showed a good fit across all used indices (*χ*^2^/df = 1.8; RMSEA = 0.057 (90% CI 0.046–0.068); CFI = 0.951; and TLI = 0.937) except a significant Chi-square (*χ*^2^ (137) = 251.276, *p*-value< 0.001). The reliability analysis of the self-efficacy scale of the HBSQ adapted for Arabic-speaking young adults is shown in Table 2, the Cronbach alpha values showed moderate to high internal consistency overall and across all subscales.

**Table 2:** Perceived self-efficacy subscales in the Arabic adaptation of the Health Beliefs Survey Questionnaire: Construct validity and Internal consistency estimates (*n* = 285)^**a**^

Item	Mean	SD	α	Skewness	Kurtosis	Item Loading
**Overall**			0.880			
**Increase fiber intake**			0.900			
Eat 1 slice of higher-fiber bread every day	57.93	35.151		−0.245	−1.136	0.858
Eat 2 slices of higher-fiber bread every day	50.19	36.638		0.113	−1.281	0.963
Eat at least 3 slices of higher-fiber bread every day	40.48	36.407		0.369	−1.181	0.794
**Decrease fat intake (1)**			0.846			
Switch to low-fat or fat-free ice cream or frozen yogurt	60.90	35.090		−0.385	−1.045	0.806
Switch to low-fat or fat-free ice cream bars	54.89	36.478		−0.178	−1.266	0.964
Eat low-fat cheese	65.37	34.496		−0.554	−0.926	0.674
**Decrease fat intake Fat (2)**			0.828			
Use low-fat spreads on bread	59.20	33.677		−0.282	−0.990	0.800
Use low-fat toppings for potatoes and other vegetables	57.30	32.080		−0.207	−0.795	0.851
Use low-fat or diet salad dressing	60.77	33.507		−0.319	−0.936	0.719
**Decrease sugar consumption**			0.681			
Avoid eating sweets for dessert	46.72	31.526		0.148	−0.692	0.688
Eat fruit for dessert instead of sweets	58.01	32.251		−0.183	−0.840	0.751
**Confidence in integrating physical activity into daily routine**			0.839			
Begin increasing your step-count again if you miss a day or two	58.03	30.354		−0.193	−0.569	0.656
Park farther away to take more steps	54.18	32.760		−0.112	− 0.919	0.692
Each week, increase your daily step-count by 500 steps	46.01	30.609		0.169	−0.600	0.694
Increase your daily step-count by 500 steps each week for 8 weeks	54.80	31.701		−0.066	−0.790	0.706
Keep track of how many steps you are taking	46.84	36.418		0.109	−1.254	0.675
Make a plan to increase your daily step-count	47.09	31.754		0.104	−0.749	0.682
**Confidence in overcoming barriers to physical activity**			0.722			
You have social activities	41.96	29.877		0.196	−0.593	0.810
You have chores or errands to do	37.46	32.997		0.450	−0.816	0.702

^**a**^Self-efficacy for healthier Foods and physical activity subscales ((0 - Certain I cannot, 50 – Somewhat certain I can, 100 - Certain I can)

α - Cronbach’s alpha coefficient; item loading – factor loading from the Confirmatory Factor Analysis.

#### Reproducibility of the adapted scales of the HBSQ

We tested reproducibility of the social support and self-efficacy scales of the HBSQ for Arabic-speaking university students. As shown in Table 3, reproducibility of the perceived social support measure, based on intraclass correlation coefficients (ICCs) of the various subscales ranged from 0.666 (perceived support from family for physical activity) to 0.904 (perceived support from family to lower fat intake).

**Table 3:** Perceived social support subscales in the Arabic adaptation of the Health Beliefs Survey Questionnaire: reproducibility after a 1-month interval (*n* = 195)

Description	Subscale	# items	ICC (95% CI)	*P* value
Perceived support from family for healthier eating	Lower fat	2	0.904 (0.874 to 0.927)	< 0.001
	Higher fiber	2	0.893 (0.861 to 0.919)	< 0.001
Perceived support from friends for healthier eating	Lower fat	2	0.887 (0.853 to 0.913)	< 0.001
	Higher fiber	2	0.875 (0.838 to 0.905)	< 0.001
Perceived support from family for physical activity		2	0.666 (0.580 to 0.738)	< 0.001
Perceived support from friends for physical activity		2	0.723 (0.649 to 0.784)	< 0.001

*ICC* intraclass correlation coefficient; *CI* Confidence interval.

The reproducibility of the perceived self-efficacy of the participants based on the ICCs of the various subscales of the self-efficacy scale, as shown in Table 4, ranged from 0.808 (increase fiber, fruit and vegetable intake) to 0.997 (overcoming barriers to physical activity).

**Table 4:** Perceived self-efficacy subscales in the Arabic adaptation of the Health Beliefs Survey Questionnaire: reproducibility after a 1-month interval (*n* = 195)

Description	Subscale	# items	ICC (95% CI)	*P* value
Confidence across time and situations	Reduce fat intake	6	0.838 (0.790 to 0.875)	< 0.001
	Reduce sugar intake	2	0.830 (0.780 to 0.869)	< 0.001
	Increase fiber, fruit and vegetable intake	3	0.808 (0.753 to 0.852)	< 0.001
Confidence in overcoming barriers	Overcoming barriers to physical activity	2	0.997 (0.996 to 0.998)	< 0.001
	Integrating physical activity into daily routine	6	0.848 (0.803 to 0.883)	< 0.001

*ICC* intraclass correlation coefficient; *CI* Confidence interval.

**Fig. 1:**
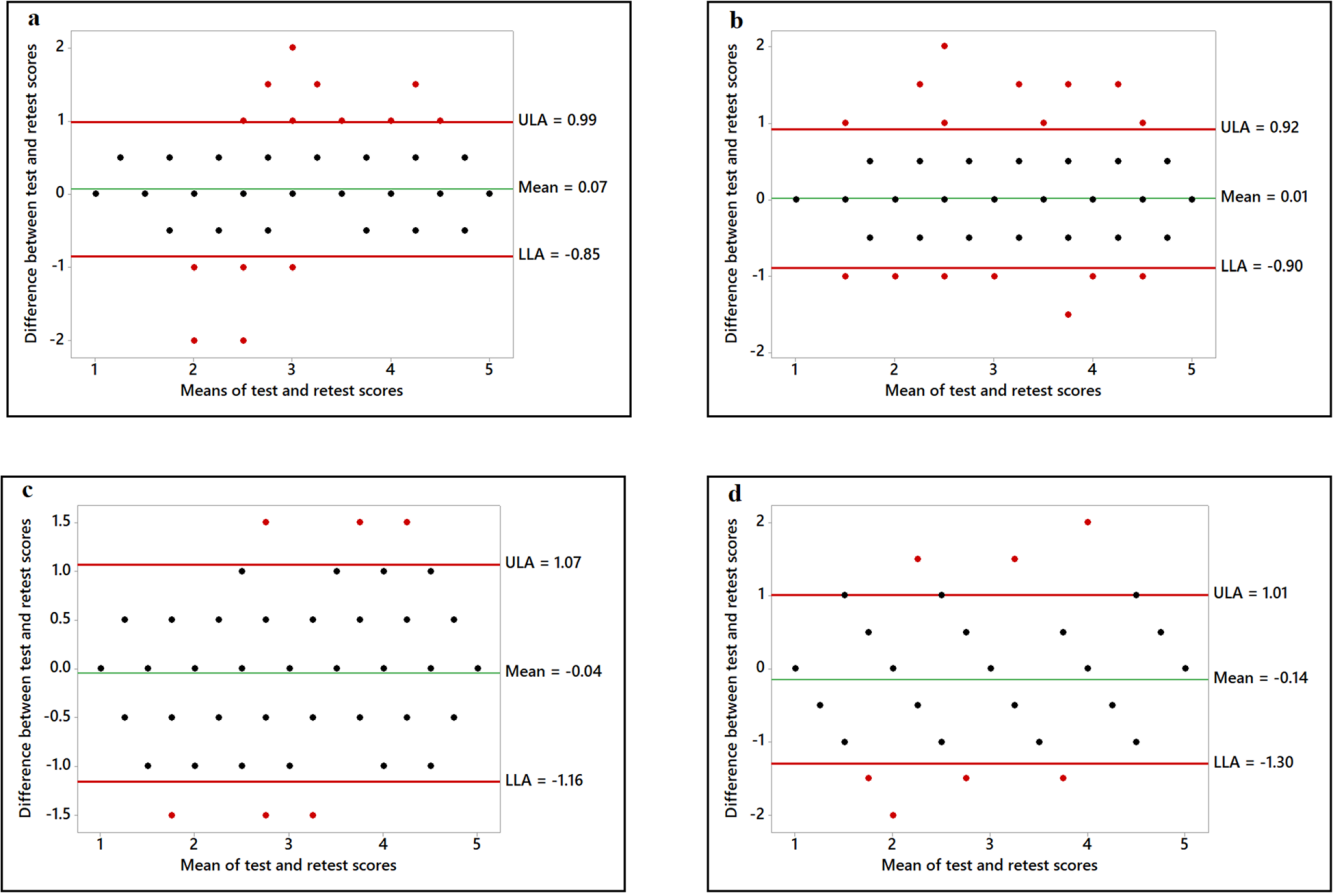
Perceived support from family and closest friends for healthy eating. Family support for decreasing fat intake (Panel **a**); Friends support for decreasing fat intake (Panel **b**); Family support for increasing fiber intake (Panel **c**); Friends support for increasing fiber intake (Panel **d**)

**Fig. 2:**
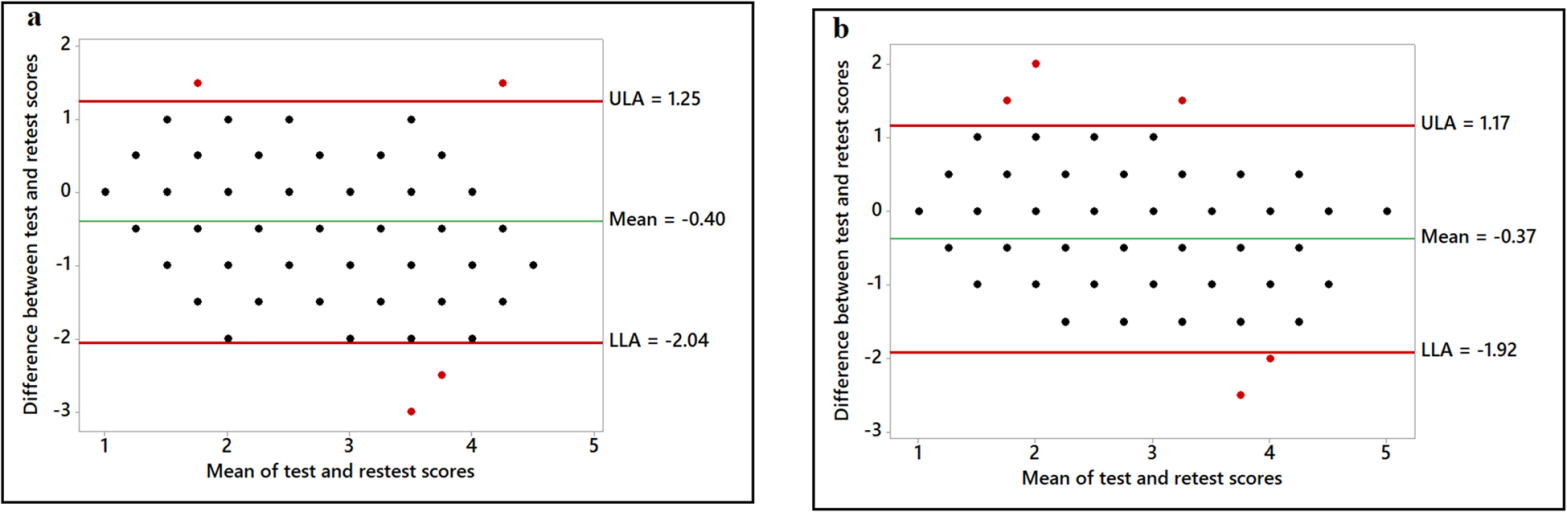
Perceived support from family and friends for engaging in regular physical activity. Family support for engaging in regular physical activity (Panel **a**); Friends support for engaging in regular physical activity (Panel **b**)

**Fig. 3:**
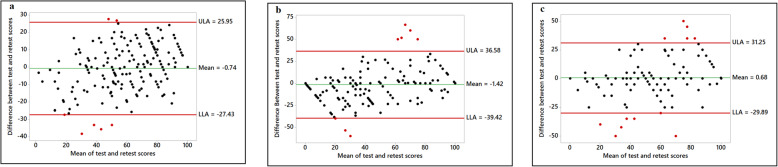
Confidence across time and situations. Self-efficacy to decrease fat intake (Panel **a**); Self-efficacy to reduce sugar intake (Panel **b**); Self-efficacy to increase fiber intake (Panel **c**)

**Fig. 4:**
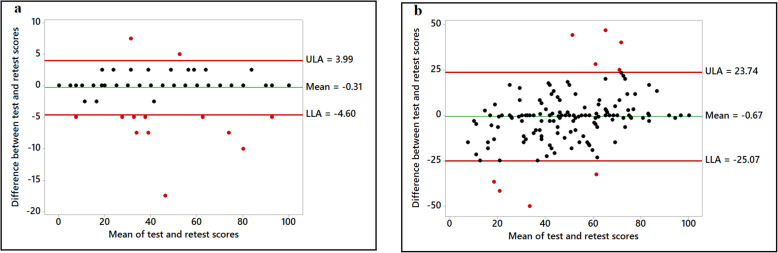
Confidence in overcoming barriers to engaging in regular physical activity. Overcoming barriers to engaging in regular physical activity (Panel **a**); Integrating physical activity into daily routine (Panel **b**)

Figures 1, 2, 3, 4 show the various Bland-Altman plots of the social support and self-efficacy subscales indicating the agreement of the responses from participants between the first and the second administration of the HBSQ adapted for Arabic-speaking university students. The differences and averages of the first and the second scores of each subscale are graphed with the centerline and the limits of agreement (LOA). Plotted points beyond the LOA were classified as outliers. As shown in Figs. 1, 2, 3, 4, Bland-Altman plots showed very good agreement between the first test and retest after a 1-month interval with very few outliers in the responses from the 195 participants. Figure 1 shows Bland-Altman plots for perceived support from family and closest friends for healthy eating by decreasing fat intake and increasing fiber, fruit and vegetable consumption. Figure 2 shows perceived support from family and friends for engaging in regular physical activity, and Fig. 3 shows the confidence of the participants across various situations to adopt healthier eating (self-efficacy to decrease fat intake, increase fiber, fruit, and vegetable intake and reduce sugar consumption). Figure 4 shows confidence in overcoming barriers to engaging in regular physical activity.

### Stage 2. Assessing perceived social support and self-efficacy levels in adopting healthy eating and engaging in regular physical activity

#### Demographic information

In the second stage of the study, 283 female students from different colleges of the United Arab Emirates University completed the adapted online questionnaire. As shown in Table 5, nearly 80% of the participants were UAE citizens, and 65% of the participants were within healthy weight (Table 5).

**Table 5:** Demographic characteristics of stage 2 participants (*n* = 283)

Characteristic	Mean ± SD	n (%)
**Age (years**)	20.4 ± 2.04	
**Nationality**		
UAE		224 (79.2)
Other Arabs		58 (20.5)
**Residence**		
On-Campus		175 (62.5)
Off-campus		105 (37.5)
**BMI (kg/m**^**2**^**)**	24.150 ± 4.66	
**BMI group**		
Normal (18.5–25.9)		184 (65)
Overweight (25.0–29.9)		70 (24.7)
Obese (≥30.0)		29 (10)
**Percent Body Fat (%)**	26.308 ± 8.59	
**Lean mass (kg)**	41.581 ± 4.08	
**Fat free mass (kg)**	43.74 ± 4.29	

#### Perceived social support to adopt healthy eating and increase physical activity

Participants reported significantly higher social support from their family members compared to their closest friends in reducing fat and increasing fiber intake (*p* < 0.001) as shown in Table 6. Based on a scale from 1 to 5 (Strongly Disagree – Agree – Neutral – Agree – Strongly Agree), the mean scores of perceived support from family and friends were 3.37 vs. 2.94, respectively, for reducing fat and 3.31 vs 2.88 for increasing fiber intake. On the other hand, there was no significant difference between perceived support from family and friends on increasing physical activity levels. We also examined whether there was a difference in the support reported by participants who were overweight or obese and those who were within normal weight. The results showed no significant difference in the perceived social support from family members or friends reported based on body weight status.

**Table 6:** Perceived social support from families and friends toward healthy eating and increased physical activity among university students (*n* = 283) ^**‡**^

Subscale^**a**^	All Participants^§^ (n = 283)	Normal Weight^§^ (*n* = 184)	Overweight or Obese^§^ (*n* = 99)	*p* value*
Reduce fat				
Family	3.37 ± 0.98 ^a^	3.35 ± 0.99 ^a^	3.37 ± 0.90 ^a^	0.862
Friends	2.94 ± 0.88 ^b^	2.91 ± 0.88 ^b^	3.01 ± 0.75 ^b^	0.372
Increase fiber				
Family	3.31 ± 0.84 ^a^	3.36 ± 0.84 ^a^	3.25 ± 0.83 ^a^	0.332
Friends	2.88 ± 0.71 ^b^	2.91 ± 0.73 ^b^	2.89 ± 0.65 ^b^	0.883
Increase physical activity				
Family	2.77 ± 0.79 ^ab^	2.70 ± 0.78 ^ab^	2.90 ± 0.73 ^ab^	0.067
Friends	2.73 ± 0.74 ^ab^	2.66 ± 0.73 ^ab^	2.85 ± 0.73 ^ab^	0.066

^**‡**^Perceived social Support (Family and Friends) for healthier foods and physical activity sub-scales (1 - Strongly disagree, 5 - Strongly agree)

Different superscripts indicate significant difference between social support subscale scores from family and friends (*p* < 0.05).

^§^All values are presented as mean ± SD

*P value < 0.05 indicates significant difference in subscale scores between BMI groups (independent t-test)

#### Perceived self-efficacy to adopt healthy eating and increase physical activity

Regarding self-efficacy and healthy eating, we found the highest mean score for reducing fat consumption and the lowest for reducing sugar intake as shown in Table 7. Based on a scale of 0 to 100 (0 – Certain I cannot, 50 – Somewhat certain I can, 100 - Certain I can), the mean score for reducing fat consumption for all participants was 60.5 vs 50.2 for reducing sugar intake. Moreover, participants were in the “somewhat certain I can” range for both integrating physical activity into their daily routine and overcoming barriers to physical activity. Finally, we examined whether there was a difference in the perceived self-efficacy of the participants who were overweight or obese and those who were within normal weight. As can be seen in Table 7, the results showed no significant difference in the perceived self-efficacy scores between the two groups.

**Table 7:** Perceived self-efficacy scores toward healthy eating and increased physical activity among normal weight and overweight/obese participants^**‡**^

Subscale^**a**^	Overall^§^ (*n* = 283)	Normal^§^ (n = 184)	Overweight/Obese^§^ (*n* = 99)	*p* value*
Reduce Fat	60.45 ± 25.71	58.24 ± 26.57	62.26 ± 23.21	0. 266
Increase fruit, vegetable and fiber intake	58.39 ± 30.62	59.24 ± 30.53	56.43 ± 29.39	0.508
Reduce sugar intake	50.18 ± 34.79	47.74 ± 35.06	50.48 ± 33.81	0.574
Integrate physical activity into daily routine	54.42 ± 32.18	55.57 ± 30.58	50.71 ± 32.96	0.270
Overcome barriers to physical activity	56.72 ± 26.80	55.13 ± 26.73	57.98 ± 27.85	0.454

^**‡**^Perceived self-Efficacy for healthier Foods and physical activity subscales (**(**0 - Certain I cannot, 50 – Somewhat certain I can, 100 - Certain I can)

*P value < 0.05 indicates a significant difference in subscale scores between BMI groups (independent t-test)

^§^All values are presented as the mean ± SD

## Discussion

The food choices of university students often do not meet recommendations. Studies conducted among university students in the UAE reported a high prevalence of overweight, obesity and unhealthy food choices [10, 11, 13, 34]. Psychosocial factors are considered key factors affecting the health behaviors of young adults [35–38]. Both self-efficacy and social support influence the adoption of healthy food choices and regular physical activity among university students [38, 39].

Social cognitive theory provides a framework for interpreting why people acquire and maintain healthful behaviors. Although the literature presents psychosocial determinants in a variety of contexts and behaviors, there is a lack of SCT-based assessment tools that measure behaviors that are related to nutrition and physical activity among Arabic-speaking young adults. In the first stage of this study, we performed successive steps to translate and adapt the social support and self-efficacy scales of the HBSQ [18] into Arabic and conducted psychometric testing of the translated questionnaire. Subsequently, we used the adapted questionnaire to assess the levels of perceived social support and self-efficacy of university students.

Two Confirmatory factor analysis (CFA) models were created, one for the social support scale and the other for self-efficacy scale. Both models had good fit based on x^2^/df, RMSEA, CFI and TLI. However, both models had significant chi square values. A potential reason for this is that Chi-square is influenced by sample size and it is almost always expected to be significant with large samples [40]. After CFA, our questionnaire had 6 items in the social support scale and 19 items in the self-efficacy scale. In comparison, the validated original English version of the HBSQ had 18 items in the social support scale and 19 items in the self-efficacy scale [28].

To our knowledge this is first study that has evaluated the model fit of a shorter Arabic version of social support and self-efficacy scales. The fit indices of the revised scales obtained using CFA were comparable to values reported by previous studies that used the full version of the scales [18, 29, 31]. The model fit demonstrated in the current study achieved using the revised shorter Arabic version of the scales might suggest that the dimensionality of the survey is retained and thus would provide better applicability and easier handling, therefore, supporting the use of the revised shorter scales and subscales. Furthermore, based on the factorial analysis, the items retained are considered the most relevant for perceived social support and self-efficacy among Arabic-Speaking female university students in the UAE. For example, items related to high fiber bread were found to be the most relevant when assessing perceived social support related to fiber intake. We also found that the most relevant items for assessing perceived social support to decrease fat intake were items related to intake of dairy products. Thus, intervention programs should target these variables in increasing social support for intake of fiber and reducing fat intake. Anderson and colleagues found that participants had healthier food purchases and intake when they perceived their family members making attempts at healthier food choices [18]. Future studies should be carried out to determine if results from Arabic-speaking female university students are consistent with this finding.

Regarding the perceived social support for physical activity, items related to the hot weather as a barrier to physical activity and use of baby sitters to increase physical activity were retained in the model both of which are highly relevant for the UAE where this study was conducted. On the other hand, the perceived social support model that was confirmed in the current student consists of two items for each of the three dimensions of the scale. One explanation is the difference in demographics of the participants in the present study and the sample used to develop the original Health Beliefs Survey Questionnaire in the United States. Future studies involving Arabic-speaking female university students in other countries are needed to confirm these findings.

The shortened HBSQ adapted for Arabic-speaking populations showed acceptable reliability based on internal consistency compared to the original English questionnaire and thus could serve as a useful tool in assessing perceived social support and self-efficacy for healthy eating and increased physical activity. Moreover, the Bland-Altman plots showed a mean difference line close to zero and lower and upper limits close to the mean difference. These results indicate the close agreement of the participant’s responses between the first and the second administration of the adapted questionnaire. Furthermore, there were few outliers above and below the mean difference.

Compared to the original HBSQ [18], the reliability of the subscale of perceived social support in the present study was slightly lower for healthy eating (0.88–0.89 vs 0.76–0.77). On the other hand, the reliabilities of the subscale of social support to increase physical activity in the original questionnaire [28] was lower than the current study (0.68 and 0.89, respectively).

The reliability of the self-efficacy measure for healthy foods measured in the original questionnaire [28] ranged from 0.76 to 0.90, while in our study, it ranged from 0.68 to 0.90. Meanwhile, the reliability of the self-efficacy for engaging in regular physical activity measure in the original study [28] was 0.89, while in our study, it ranged from 0.72–0.84. Although some similarities and differences in the reliability of the original and the adopted HBSQ were observed, there are several possible reasons for these slight differences that should be mentioned. Populations and demographics were different in the two studies. In the study of Anderson and colleagues [18], the participants were older, 18% African American, 66% female, and 79% overweight or obese and were recruited from 14 churches in Southwestern Virginia, USA. On the other hand, the participants in our questionnaire adaptation study were much younger than those who completed the original questionnaire (mean age: 53.54 vs 21.4 years).

Participants in the second stage of our study reported higher perceived social support from their families compared to their friends regarding reducing fat intake and increasing fiber intake. Therefore, intervention strategies targeting the enhancement of social support from friends should be considered. This may include creating online groups with their peers that are guided by a trained coach to assist university students in improving their food choices and physical activity levels. Moreover, intervention strategies for college students should incorporate strategies designed to increase the support of health-promoting activities from their families. The low scores of all the self-efficacy subscales (ranges 50–61 out of maximum rating of 100) indicate that university students in the UAE have low self-efficacy irrespective of their weight status.

The main limitation of this study is that it included a convenience sample of female university students from a single university setting. Moreover, previous research has shown gender differences in relation to diet, physical activity and body image [41–43]. This could limit the generalizability of the study findings. However, the sample included students from various Arabic-speaking countries. Future research with a sample drawn from wider geographic areas and demographic backgrounds needs to be conducted to adapt the questionnaire for a wider segment of Arabic-speaking populations. On the other hand, this is the first study that has evaluated the adoption of the HBSQ for Arabic-speaking populations. This tool will be useful in both assessing the social support and self-efficacy of Arabic-speaking populations as well as evaluating the impact of interventions designed to enhance social support and self-efficacy of research participants. Moreover, for the first time to our knowledge, we used an SCT-based questionnaire to assess the perceived social support and self-efficacy levels of Arabic-speaking university students. The findings of this study have implications for designing intervention programs promoting healthy eating and physical activity among university students in the UAE as well as stimulating further research in this area.

## Conclusions

Our results indicate that the shorter and revised Arabic version of the HBSQ is a reliable and useful tool to assess perceived social support from family and close friends as well as perceived self-efficacy for choosing healthy foods and increasing physical activity among Arabic-speaking female university students. Although the scales were tested among female university students, they might be applicable to other Arabic-speaking young adults. However, this should be confirmed with further studies. The low scores in perceived social support and self-efficacy scales, reported in the second stage of the study, suggest that future interventions should consider increasing support from family and friends as well as improving self-efficacy related to healthy eating and physical activity to help young adults adopt healthy lifestyle behaviors.

## Data Availability

The datasets used and/or analyzed during the current study are available from the corresponding author on reasonable request.

## Abbreviations

HBSQ: Health beliefs survey questionnaire
CFA: Confirmatory factor analysis
UAE: United Arab Emirates
SCT: Social cognitive theory
LOA: Limits of agreement
ICC: Intraclass CORRELATION COEFFICIENT
RMSEA: Root mean square error of approximation
CFI: Comparative fit index
TLI: Tucker-lewis index

## References

[1] Ng SW, Zaghloul S, Ali HI, Harrison G, Popkin BM (2011). The prevalence and trends of overweight, obesity and nutrition-related non-communicable diseases in the Arabian Gulf States. Obes Rev.

[2] Hajat C, Harrison O, Shather Z (2012). A profile and approach to chronic disease in Abu Dhabi. Glob Health.

[3] Loney T, Aw TC, Handysides DG, Ali R, Blair I, Grivna M, Shah SM, Sheek-Hussein M, el-Sadig M, Sharif AA, el-Obaid Y (2013). An analysis of the health status of the United Arab Emirates: the 'Big 4′ public health issues. Glob Health Action.

[4] Ng SW, Zaghloul S, Ali H, Harrison G, Yeatts K, El Sadig M (2011). Nutrition transition in the United Arab Emirates. Eur J Clin Nutr.

[5] Imamura F, Micha R, Khatibzadeh S, Fahimi S, Shi P, Powles J, Mozaffarian D, Global Burden of Diseases Nutrition and Chronic Diseases Expert Group (NutriCoDE) (2015). Dietary quality among men and women in 187 countries in 1990 and 2010: a systematic assessment. Lancet Glob Health.

[6] Papadaki A, Hondros GA, Scott J, Kapsokefalou M (2007). Eating habits of university students living at, or away from home in Greece. Appetite.

[7] Racette SB, Deusinger SS, Strube MJ, Highstein GR, Deusinger RH (2005). Weight changes, exercise, and dietary patterns during freshman and sophomore years of college. J Am Coll Heal.

[8] Winpenny EM, van Sluijs EMF, White M, Klepp KI, Wold B, Lien N (2018). Changes in diet through adolescence and early adulthood: longitudinal trajectories and association with key life transitions. Int J Behav Nutr Phys Act.

[9] Kerkadi A (2003). Evaluation of nutritional status of united Arab Emirates university female students. Emir J Food Agric.

[10] Al SH (2020). Prevalence of overweight/obesity, anaemia and their associations among female university students in Dubai, United Arab Emirates: a cross-sectional study. J Nutr Sci.

[11] Al Dhaheri AS, Al Maawali AK, Laleye LC, Washi S (2014). Nutritional knowledge of Emirati traditional foods and body image perceptions among UAE University students. Emir J Food Agric.

[12] Radwan H, Hasan HA, Ismat H, Hakim H, Khalid H, Al-Fityani L (2019). Body mass index perception, body image dissatisfaction and their relations with weight-related behaviors among university students. Int J Environ Res Public Health.

[13] Khawaja AH, Qassim S, Hassan NAGM, Arafa E-SA (2019). Added sugar: nutritional knowledge and consumption pattern of a principal driver of obesity and diabetes among undergraduates in UAE. Diabetes Metab Syndr Clin Res Rev.

[14] Karfopoulou E, Anastasiou CA, Avgeraki E, Kosmidis MH, Yannakoulia M (2016). The role of social support in weight loss maintenance: results from the med weight study. J Behav Med.

[15] Munt AE, Partridge SR, Allman-Farinelli M (2017). The barriers and enablers of healthy eating among young adults: a missing piece of the obesity puzzle: a scoping review. Obes Rev.

[16] Ali HI, Jarrar AH, Abo-El-Enen M, Al Shamsi M, Al AH (2015). Students' perspectives on promoting healthful food choices from campus vending machines: a qualitative interview study. BMC Public Health.

[17] Bandura A (1986). Social foundations of thought and action: a social cognitive theory.

[18] Anderson ES, Winett RA, Wojcik JR (2007). Self-regulation, self-efficacy, outcome expectations, and social support: social cognitive theory and nutrition behavior. Ann Behav Med.

[19] Dewar DL, Lubans DR, Morgan PJ, Plotnikoff RC (2013). Development and evaluation of social cognitive measures related to adolescent physical activity. J Phys Act Health.

[20] Ramirez E, Kulinna PH, Cothran D (2012). Constructs of physical activity behaviour in children: the usefulness of social cognitive theory. Psychol Sport Exerc.

[21] Dai CL, Sharma M (2015). Predicting childhood obesity prevention behaviors using social cognitive theory for elementary school students in Taiwan. Int J Health Promot Educ.

[22] Bandura A (1977). Self-efficacy: toward a unifying theory of behavioral change. Psychol Rev.

[23] Bandura A (1997). Self-efficacy: the exercise of control.

[24] Craddock E, van Dellen MR, Novak SA, Ranby KW (2015). Influence in relationships: a meta-analysis on health-related social control. Basic Appl Soc Psychol.

[25] Deliens T, Clarys P, De Bourdeaudhuij I, Deforche B (2014). Determinants of eating behaviour in university students: a qualitative study using focus group discussions. BMC Public Health.

[26] Ford ES, Ahluwalia IB, Galuska DA (2000). Social relationships and cardiovascular disease risk factors: findings from the third national health and nutrition examination survey. Prev Med.

[27] Steptoe A, Perkins-Porras L, Rink E, Hilton S, Cappuccio FP (2004). Psychological and social predictors of changes in fruit and vegetable consumption over 12 months following behavioral and nutrition education counseling. Health Psychol.

[28] Anderson ES, Winett RA, Wojcik JR, Williams DM (2010). Social cognitive mediators of change in a group randomized nutrition and physical activity intervention. J Health Psychol.

[29] Anderson ES, Winett RA, Wojcik JR (2000). Social-cognitive determinants of nutrition behavior among supermarket food shoppers: a structural equation analysis. Health Psychol.

[30] Anderson ES, Wojcik JR, Winett RA, Williams DM (2006). Social-cognitive determinants of physical activity: the influence of social support, self-efficacy, outcome expectations, and self-regulation among participants in a church-based health promotion study. Health Psychol.

[31] Anderson ES, Winett RA, Wojcik JR, Winett SG, Bowden T (2001). Social cognitive intervention for nutrition behavior: direct and mediated effects on fat, fiber, fruits, and vegetables, self-efficacy, and outcome expectations among food shoppers. Ann Behav Med.

[32] World Health Organization (WHO). Process of translation and adaptation of instruments. 2020. https://www.who.int/substance_abuse/research_tools/translation/en/#. Accessed 20 Oct 2020.

[33] Xia Y, Yang Y (2019). RMSEA, CFI, and TLI in structural equation modeling with ordered categorical data: the story they tell depends on the estimation methods. Behav Res Methods.

[34] Al Dhaheri AS, Mohamad MN, Jarrar AH, Ohuma EO, Ismail LC, Al Meqbaali FT (2016). A cross-sectional study of the prevalence of metabolic syndrome among young female emirati adults. PLoS One.

[35] Farren GL, Zhang T, Martin SB, Thomas KT (2017). Factors related to meeting physical activity guidelines in active college students: a social cognitive perspective. J Am Coll Heal.

[36] Lim HJ, Kim MJ, Kim KW (2015). Factors associated with nutrition label use among female college students applying the theory of planned behavior. Nutr Res Pract.

[37] Menozzi D, Sogari G, Mora C (2015). Explaining vegetable consumption among young adults: an application of the theory of planned behaviour. Nutrients..

[38] Sogari G, Velez-Argumedo C, Gómez MI, Mora C (2018). College students and eating habits: a study using an ecological model for healthy behavior. Nutrients..

[39] Kim MJ, Kim KW (2015). Nutrition knowledge, outcome expectations, self-efficacy, and eating behaviors by calcium intake level in Korean female college students. Nutr Res Pract.

[40] Sun J (2005). Assessing goodness of fit in confirmatory factor analysis. Meas Eval Couns Dev.

[41] Landry M, Lemieux S, Lapointe A, Bédard A, Bélanger-Gravel A, Bégin C, Provencher V, Desroches S (2018). Is eating pleasure compatible with healthy eating? A qualitative study on Quebecers' perceptions. Appetite..

[42] Heiman T, Olenik-Shemesh D (2019). Perceived body appearance and eating habits: the voice of young and adult students attending higher education. Int J Environ Res Public Health.

[43] El Ansari W, Dibba E, Stock C (2014). Body image concerns: levels, correlates and gender differences among students in the United Kingdom. Cent Eur J Public Health.

